# The Effects of H_2_S and Recombinant Human Hsp70 on Inflammation Induced by SARS and Other Agents In Vitro and In Vivo

**DOI:** 10.3390/biomedicines10092155

**Published:** 2022-09-01

**Authors:** Sergei Onikienko, Maxim Vinokurov, Marina Yurinskaya, Alexander Zemlyanoi, Sergei Abkin, Elvira Shaykhutdinova, Victor Palikov, Alexander Ivanov, Olga Smirnova, Irina Fedyakina, Natalia Bychkova, Olga Zatsepina, David Garbuz, Michael Evgen’ev

**Affiliations:** 1 Saint-Petersburg Scientific Center, RAS (Russian Academy of Sciences), 199034 St. Petersburgh, Russia; 2 Institute of Cell Biophysics, RAS (Russian Academy of Sciences), 142290 Pushchino, Russia; 3 Institute of Bioorganic Chemistry, RAS (Russian Academy of Sciences), 142290 Pushchino, Russia; 4 Engelhardt Institute of Molecular Biology, RAS (Russian Academy of Sciences), 119991 Moscow, Russia; 5 The Nikiforov Russian Centre of Emergency and Radiation Medicine, 194044, St. Petersburg, Russia

**Keywords:** inflammation, LPS-challenge, sodium thiosulfate, rHsp70, pneumonia, COVID-19

## Abstract

The ongoing epidemic caused by SARS-CoV-2 infection led to the search for fundamentally new ways and means to combat inflammation and other pathologies caused by this virus. Using a cellular model of lipopolysaccharide (LPS)-induced sepsis (human promonocytes), we showed that both a hydrogen sulfide donor (sodium thiosulfate, STS) and a recombinant Heat shock protein 70 (rHsp70) effectively block all major inflammatory mediators when administrated before and after LPS challenge. The protective anti-inflammatory effect of rHsp70 and H_2_S was also confirmed in vivo using various animal models of pneumonia. Specifically, it was found that rHsp70 injections prevented the development of the acute respiratory distress syndrome in highly pathogenic pneumonia in mice, increased animal survival, and reduced the number of Programmed death-1 (PD-1)-positive T-lymphocytes in peripheral blood. Based on our model experiments we developed a combined two-phase therapeutic approach for the treatment of COVID-19 patients. This procedure includes the inhalation of hot helium–oxygen mixtures for induction of endogenous Hsp70 in the first phase and STS inhalation in the second phase. The use of this approach has yielded positive results in COVID-19 patients, reducing the area of lung lesions, restoring parameters of innate immunity and T-cell immune response against coronavirus infection, and preventing the development of pulmonary fibrosis and immune exhaustion syndrome.

## 1. Introduction

The ongoing COVID-19 epidemic caused by the highly infectious and deadly coronavirus (SARS-CoV-2) that causes acute respiratory syndrome [1], which has affected all countries and killed over 6 million people, has presented the scientific community with several priorities that require coordinated work by medical and scientific experts in a variety of fields. Despite the vaccines of different nature adopted by the Food and drug Administration (FDA) and widely used all over the world, it is already clear that this quickly mutating virus and its derivatives will remain the main threat to humanity in the coming decades. The development and timely use of antivirus therapy at different stages of the disease and in rehabilitation is highly relevant and requires both an understanding of the molecular mechanisms of infection by this virus and the human body’s response at the cellular and molecular level to viral and bacterial infection. As numerous studies have shown [2,3,4,5], that one of the main manifestations of SARS-CoV-2 infection is an excessive response of the body’s immune system, including macrophage activation and “cytokine storm”, leading to acute lung inflammation. In this case, there is a sharp increase in the levels of major cytokines (IL-6, IL-1, TNF-a, etc.), as well as the development of acute respiratory distress syndrome (ARDS), disseminated intravascular coagulation syndrome, and multiple organ failure, as well as septic shock, which often leads to fatal consequences. Therefore, there is an urgent need for non-toxic drugs capable of preventing and/or suppressing cytokine storm and other pathological manifestations of cell damage by this virus. As promising candidates for the role of such protective antiviral agents, we suggest a harmless hydrogen sulfide (H_2_S) donor, sodium thiosulfate (STS), and a recombinant human stress protein (rHsp70), that can ameliorate viral infection at different stages.

The 70 kDa heat shock protein is the most versatile of the chaperone proteins responsible for the folding of proteins synthesized in the cell, refolding proteins partially denatured by various stresses, including heat shock and viral infections [6,7,8]. It was shown that secreted Hsp70 is recognized by several so-called pattern-recognizing receptors, which are a crucial link in the innate immunity system. These are TLR2 and TLR4, that belong to the Toll-like receptors group, as well as lectin-like oxidized low-density lipoprotein receptor LOX-1, scavenger receptor SREC-1, and CLEVER-1 receptors (scavenger receptors group, SR). The term “chaperokine” was suggested for exogenous Hsp70, emphasizing the dichotomy of Hsp70 functions: as a molecular chaperone (intracellular form) and a transmitter of intercellular signals, realized through a specific ligand-receptor interaction, similar to the classical cytokines. One of the natural TLR4 ligands is the secreted form of Hsp70. Many studies have shown that recombinant Hsp70 has an anti-inflammatory effect and inhibits the expression of major inflammatory mediators. Thus, the administration of rHsp70 blocks the production of TNFα (Tumor necrosis factor α) and ROS (Reactive Oxygen Species) by neutrophils and macrophages in response to various bacterial toxins [9,10,11,12]. Interaction of rHsp70 with TLR4 leads to rapid phagocytosis of the formed complex [13]. Endogenous, intracellular Hsp70 is also known to inhibit TLR4 activity, contributing to its ubiquitin-dependent degradation [14]. In SARS-CoV-2 infection, spike virus envelope protein, such as LPS, has been shown to interact with TLR4, leading to the synthesis of proinflammatory cytokines [2,15] and the occurrence of a “cytokine storm”.

Another important anti-inflammatory agent is hydrogen sulfide (H_2_S), which in recent years has taken its rightful place among other important gas transmitters (i.e., CO and NO) and has received widespread attention from both scientists and physicians [4,15,16,17,18,19]. H_2_S is formed in cells as a result of the transsulfuration process described in almost all prokaryotic and eukaryotic organisms, including humans [19]. Disturbances in the functioning of this system, which includes three major genes involved in H_2_S production (*cbs*-*cystathionine beta synthase*, *cse*-*cystathionine* *γ-lyase*, and *mst-mercaptopyruvate sulfurtransferase*), lead to a variety of pathologies, including cancer, cardiovascular, and neurodegenerative diseases [2,4,20]. Although H_2_S is known to be poisonous at high concentrations, at low concentrations it protects cells from oxidative stress. Hydrogen sulfide donors, particularly sodium thiosulfate (STS), have long been used successfully in medicine for cyanide poisoning, calciphylaxis, pulmonary inflammation, heart disease, and several other human diseases [2,4,21,22,23,24,25,26]. In recent years, it has been found that patients with high endogenous hydrogen sulfide levels in the case of COVID-19 infection have milder disease and significantly lower mortality rates [2,27].

In recent decades, our laboratory has conducted comprehensive studies of the protective properties of recombinant human Hsp70 and various H_2_S donors at the cellular and organismal (mice, rats, *Drosophila*) levels [6,11,12,23,28,29,30]. Our studies showed that both H_2_S donors and rHsp70 show a significant anti-inflammatory effect in all the model systems we used (cell cultures, model organisms), reducing the level of the main inflammatory mediators. Importantly, the rHsp70 we used also exhibited protective, anti-inflammatory properties in animal models of various neurodegenerative diseases, including Alzheimer’s disease, as well as models of sepsis and stroke [11,31,32]. As far as we know, there are no data on the combined use of exogenous Hsp70 and hydrogen sulfide donors such as STS to protect human and animal cells from bacterial endotoxins and viruses. Interestingly, there is evidence that increased levels of H_2_S in the cell can lead to induction of Hsp70 synthesis [33,34]. In the present work, we investigated the anti-inflammatory effects of rHsp70 and a harmless, FDA-approved H_2_S donor (STS) on LPS-exposed human monocyte cell culture (THP1). In addition, the protective effects of rHsp70 and STS on various models of lung inflammation, including COVID-19 pneumonia in humans, were investigated. The method we developed, which explores inhalation of hot oxygen–helium mixtures and STS sequentially, demonstrated reliable positive results when used in COVID-19 patients, effectively blocking inflammation, restoring innate and adaptive immunity, and preventing the development of fibrosis in the lungs.

## 2. Materials and Methods

### 2.1. The Isolation of Recombinant Human Hsp70

We previously described in detail the preparation and purification of human recombinant HSP70 (rHsp70) [31,32]. The Fc-Hsp70 was expressed as a fusion of human rHsp70 and the Fc fragment of immunoglobulin as the N-terminal fusion partner [35]. A method to obtain and purify rHsp70-Fc, which has a significantly longer lifetime than the original rHsp70, is given in patent US 9,217,018 [36].

### 2.2. Cell Lines and Their Cultivation

Human promonocytic cell line, THP-1, was obtained from the American Type Culture Collection (ATCC, Manassas, USA). The cells were cultured in a RPMI-1640 medium (SigmaAldrich, St.Louis, MO, USA) containing 10% heat-inactivated FCS, 2 mM L-glutamine, 100 units/mL of penicillin, and 100 μg/mL streptomycin at 37 °C under 5% CO_2_. For differentiation, THP-1 cells in the culture medium were placed into 24-well flat-bottomed plates, and 200 nM phorbol-12-myristate-13-acetate was added. The cells were grown for 72 h at 37 °C under 5% CO_2_, washed with culture medium and were subsequently used in the experiments. Thereafter, the cells were supplemented with STS (0.2 μM–2 mM) or 200 μM GYY4137, (for 120 min), and then 1 μg/mL LPS *E.coli* (serotype O55:B5) was added to the corresponding samples and the cells were incubated for 24 h at 37 °C under 5% CO_2_.

L-929 cells were obtained from the Russia Tissue Culture Collection, Institute of Cytology of the Russian Academy of Science.

The Vero E6 cells derived from green monkey kidney were from the Russian National Collection of Cell Cultures at the National Research Center for Epidemiology and Microbiology named after Honorary Academician N.F. Gamaleya of the Ministry of Health of the Russian Federation (Moscow, Russia). The cells were checked for mycoplasma contamination by standard PCR every two weeks. All cells were cultivated at 37 °C in a humid atmosphere containing 5% CO_2_. The standard media were supplemented with 10% fetal bovine serum (FBS) and 2 mM glutamine if not stated otherwise. As a standard medium, we used DMEM supplemented with 2% FBS for Vero E6 cells. Twenty-four hours before analysis, cells were seeded on 6-well-plates at a density of 1.8 × 10^4^ cells/well.

### 2.3. The Determination of NO, ROS, TNFα, H_2_S Levels in the Cell Lines

#### 2.3.1. Measurement of Nitric Oxide

The level of nitrites was estimated using a Griess reagent (Sigma, St. Louis, MO, USA), described in [28,29]. Briefly, after cell cultivation, 100 µL aliquots of each culture supernatant were placed into 96-well flat-bottomed plates. Griess reagent was added to each well, and the mixture was incubated for 10 min at room temperature in the dark. The absorbance at 540 nm was determined using a Uniplan plate reader. Triplicate wells were assayed under each condition, and the standard deviation was determined.

#### 2.3.2. Determination of ROS Level with NBT

ROS production in the cells was estimated using nitro-blue tetrazolium (NBT) [28,29]. At the end of the cultivation, 0.1% NBT solution was added to cells and cells were incubated at 37 °C and 5% CO_2_ for 2 h. Next, the cells were washed twice with phosphate buffered saline (PBS), fixed with ethanol and dried. Intracellular formazan was dissolved in 300 μL 2 M KOH and 400 μL DMSO; optical density was determined at 620 nm using a Uniplan plate reader (ZAO PICON, Moscow, Russia).

#### 2.3.3. The Determination of the TNF-α Levels

TNF-α production was evaluated based on the cytotoxic effect of THP-1 supernatants, using L-929 cells as the target. L-929 cells (2 × 10^4^ cells/100 μL) were placed into 96-well flat-bottomed plates and were cultured in a RPMI-1640 medium containing 10% heat-inactivated FCS, 2 mM L-glutamine, 100 units/mL penicillin, and 100 μg/mL streptomycin at 37 °C under 5% CO_2_ for 24 h. After 24 h, actinomycin D (1 μg/mL) was added to the monolayer in addition to 100 μL of the supernatant from cells in each well. Pure medium was added to the control wells. The plates were incubated for 24 h at 37 °C under 5% CO2 and were stained with crystal violet. Survival of the cells was determined after the crystals were completely dissolved in 1% SDS. The absorbance at 595 nm was determined using a Uniplan plate reader. Triplicate wells were assayed for each condition and the SD was determined. The relative levels of TNF-α production were determined based on the toxicity index, as described previously [28,29].

#### 2.3.4. Intracellular H_2_S Determination

In order to monitor the production of H_2_S by both donors (GYY4137 and STS), we applied a modern technique using fluorescent compound SF7-AM (Ex/Em = 498/526 nm) essentially as described [37]. The kinetics of H_2_S production was monitored using a flow cytometry analysis (cytometer CytoFLEX S “Beckman Coulter”, Brea, CA, USA). The level of TLR4 was estimated using Anti-TLR4 (Toll-Like Receptor 4, CD284) (PE) (US Biological, T8050-34D, Salem, MA, USA), according to the manufacturer’s recommendations.

### 2.4. Statistical Analysis

The data are shown as the mean ± standard deviation in the 6 independent experiments performed as quadruplicates. The differences among the groups were analyzed by a one-way ANOVA with Tukey’s pairwise comparisons. The Shapiro–Wilk test was applied to check for normal distribution of the values being analyzed. Significance thresholds were set to * *p* < 0.05, ** *p* < 0.01, *** *p* < 0.005, **** *p* < 0.001, ^#^ *p* < 0.00.

### 2.5. Monitoring of the Effect of STS and GYY4137 on SARS-CoV-2 Replication

SARS-CoV-2 human coronavirus (passage 3) with infectivity of 10^7.5^ TCID_50_/_mL_ of hCoV-19/Russia/Moscow-PMVL-12/2020 strain was used. Vero E6 cells were seeded 24 h before infection in 6-well plates at 4 × 10^4^ cells/well density in DMEM supplemented with 2.5% FBS. When the cells reached >95% confluency, they were inoculated with the pre-diluted virus at 0.1 MOI and shacked every 20–30 min. Two hours later the medium was removed, the cells were washed with PBS, and a fresh medium containing the drugs was added. Total RNA was purified from a conditioned medium 4 days post-infection using the High Pure RNA Isolation Kit (Roche Life Sciences, Schlieren, Switzerland) according to the manufacturer’s instructions. Reverse transcription and quantification of SARS-CoV-2 genomic RNA were performed as described in [38].

The data accumulated in the above-described experiments as well as the abundant literature describing the anti-inflammation effects of H_2_S, and specifically its donor STS, motivated us to further investigate the role of this agent in the case of SARS-CoV-2 infection.

### 2.6. The Analysis of the Anti-Inflammatory Effect of STS Inhalation in LPS-Induced Pneumonia in Rats

The experiments involving LPS-challenge were carried out on adult male Wistar rats 11 to 12 weeks old (300–350 g of body weight). All animals were maintained in the two corridors barrier-type facility (animal barrier zone of BTL IBC RAS) with the automatic change of day and night time (08:00–20:00—“Day”, 20:00–08:00”—Night”) and the renewal of the room air at least 12 times hourly. The animals were randomly divided into three groups (five animals in each group, *n* = 5; see also Table S1 for Schemes): Group 1, control animals with acute respiratory distress syndrome (ARDS) and treatment of normal saline in Scheme 1 (i.e., one hour after LPS); Group 2, ARDS with the treatment of test item, sodium thiosulfate (STS) in Scheme 1; Group 3, ARDS with the administration of STS in Scheme 2 (1 h before LPS,). ARDS was induced by LPS treatment under general anesthesia (Zoletil^®^/Xyla^®^ intramuscular). A single intra-tracheal installation (through the mouth) was made with LPS (Lipopolysaccharides from *Escherichia coli* O111:B4, Sigma Aldrich, St.Louis, USA) 0.5 mL/kg (2 mg/mL) using a catheter. Then, a ventilator (UGO Baseline, Rodent Ventilator, Milan, Italy) was connected to the catheter with parameters of ventilation of 60 beats/min, and tidal volume of 30 mL/kg. The time of hyperventilation was 20 min. The test item, 5% sodium thiosulfate (STS) was inhaled for 20 min using the head-nose-only apparatus TSE System GmbH at the 2.5 mg/L nominal exposure level. The aerosol was of respirable particle size 1–4 μm (mass median aerodynamic diameter, MMAD). A control group was aerosolized by the vehicle (saline). Scheme 1 included inhalation of STS or saline after ARDS 1 h later and once daily (on Days 2 to 7). Scheme 2 included inhalation of STS 1 h before ARDS and once daily too (on Days 2 to 7). At the end of the study (Day 8), the animals were anesthetized (Zoletil^®^/Xyla^®^) and euthanized following terminal blood sampling from *vena cavae* for measuring IL-6 serum levels. The serum was separated and stored at −20 °C until analysis. According to the manufacturer’s instructions, analysis was performed to measure serum IL-6 levels using the IL-6 rat Elisa kit (Thermo Fisher Scientific, Waltham, MA, USA) and Multiskan™GO Microplate Spectrophotometer (Thermo Scientific, Japan).

### 2.7. Study of the Antifibrotic Role of Hsp70 in LPS-Induced Pneumonia in Rats

The expression of alpha-smooth muscle actin (αSMA) was evaluated to detect early signs of pulmonary fibrosis in the LPS-induced pneumonia model in Wistar rats. In this series of experiments, an intratracheal injection of LPS at a dose of 2 mg/mL in combination with an intraperitoneal injection of rHsp70 and rHsp70-Fc was performed. After modeling LPS-induced pneumonia in rats, as described above, 50 rats were divided into 6 groups, respecting individual labeling: Group #1—intact animals (*n* = 6); Group #2—control, LPS (no treatment) (*n* = 12); Group #3—LPS, administration of 100 µg/rat Hsp70 (*n* = 8); Group #4—LPS, administration of Hsp70 250 µg/rat (*n* = 8); Group No. 5—LPS, administration of Hsp70 500 µg/rat (*n* = 8); Group No. 6—LPS, administration of Hsp70-Fc 350 µg/rat (*n* = 8). Animals of the experimental groups after modeling LPS-induced pneumonia were intraperitoneally injected with the studied preparations starting from 3 days after the experiment Hsp70 daily (14 injections in total) HSP70-Fc twice a week (7 injections in total). Animals of the control group were injected intraperitoneally with a physiological solution of 0.5 mL for 14 days. The result was evaluated 28 days after the beginning of the experiment after the decapitation of the animals. Details of the histochemical analysis of lung tissues of control and experimental animals are described in Supplementary Materials. All samples were analyzed in one assay.

### 2.8. The Analysis of the Protective Role of rHsp70 in the Model of Virus-Induced Pneumonia in Mice

Pulmonary edema in Balb/c mice was induced by intranasal infection with mouse pathogenic influenza virus H3N2 (3 LD50). To protect against lethal influenza infection, rHsp70 (50 µg/mouse) was injected 10 min after infection and then daily for 4 days. Recombinant Hsp70 (50 µg/mouse) was injected intraperitoneally into mice. Human rHsp70 or rHsp70-Fc was used in this work. Animals were divided into 4 groups (*n* = 20). Group 1 animals received rHsp70, Group 2—rHsp70-Fc, Group 3 (control)—physiological solution in the same volume, Group 4—intact animals. The severity of pulmonary edema was determined by the value of lung coefficient (LC) LC = lung weight/animal weight × 1000. The number of CD3+CD8+PD1+ lymphocytes was determined in the blood plasma of mice by flow cytometry (Cell Lab Quanta ™ SC, Beckman Coulter, USA) using mouse antibodies from Biolegend (USA).

### 2.9. Human Studies

When working with COVID-19 patients and healthy volunteers, we used our original technology, leading to the induction of synthesis and release of heat shock proteins (including Hsp70) from lung cells by inhalation of helium–oxygen breathing mixtures heated to 100–120 ° C. Such hot mixtures do not cause thermal damage to target tissues. We studied the effectiveness of combined use of hot helium–oxygen mixtures (inducer of endogenous Hsp70) with inhalation administration of sodium thiosulfate (STS), a hydrogen sulfide donor, using the mesh nebulizer “Aerogen Pro”, LLC “Aerogen”, Ireland. The inclusion of a nebulizer of this type in the breathing circuit of the device for inhalation of hot helium–oxygen mixtures allows increasing significantly the efficiency of drug delivery to hard-to-reach parts of the lungs (Russian Federation patent No. 199823).

The inhalation of heated helium–oxygen breathing mixture (He-70%, O_2_-30%) is carried out for 5–10 min, and then inhalation is performed using “Thermohelix-Pro” apparatus and Pari Velox mesh nebulizer [39,40] using 5 mL of 7.5% sodium thiosulfate solution 2–3 times a day. The course of treatment is 7–14 days. In some cases, inhalation of 1 mL of 0.5–1.0% lidocaine solution for 5–10 min was used before STS application to reduce upper airway irritation sometimes caused by STS. COVID-19 infection in patients was detected using the DNA-Technology PCR test, the sensitivity of 5 × 10^2^ copies of SARS-CoV-2 RNA per mL. Antibodies to SARS-Cov-2 virus proteins (IgG, IgM) in blood serum were determined by ELISA using GA Cov-2 IgG, IgM test system manufactured by GA Generic Assays GmbH, Germany. The result was considered positive (antibodies detected) if the ELISA values > 1.1.

The number of CD3+, CD3+CD8+, CD3+CD8+PD-1+, lymphocytes in the blood was determined by flow cytometry (Cell Lab QantaTM SC “Beckman Coulter”, Brea, CA, USA), using (CD45-APC-Alexa Fluor 750; CD8-FITC; CD3-PE; PD-1-PC5.5) antibodies of Biolegend (San Diego, CA, USA).

A comprehensive assessment of T-cell immunity to COVID-19 was performed. Antibody immunity was assessed using the SARS-Cov-2-IgG-IFA-BEST test system, Vector-Best JSC. The result was considered positive if ELISA > 10 BAU/mL. T-cell immune response to COVID-19 was determined using the T-SPOT^®^.COVID test, Oxford Immunotec [41]. The result was considered positive if the number of T-lymphocyte spots to the peptide antigens of S, N, M, O3, O7 proteins of coronavirus > 12.

The effectiveness of coronavirus pneumonia treatment was assessed by determining the dynamics of lung CT scores (Philips Ingenuity 128, Vidar Dicom Viewer 3.2 image analysis software). Impairments of external respiratory function were determined by body plethysmography (Master Screen Body, Erich Jaeger, Germany).

Sixty-nine people in contact with COVID-19 patients (medical employers and relatives of the patients) participated in the study of the preventive effect of STS. Members of this group received daily inhalation of hot helium–oxygen mixtures in combination with inhalation of 7.5% sodium thiosulfate. The mean age of the participants was 29 years, of whom 62% were female. The comparison group consisted of medical employers and relatives of patients who did not receive inhalation (82 individuals). This group was analyzed retrospectively. Results were recorded on days 7, 14, and 28 of the study. The main indicator of the effectiveness of STS action is the reduction in the number of cases of coronavirus infection. The criterion for the effectiveness of the treatment was a negative PCR test and the absence of clinical signs of the disease. The detailed description of both groups is provided in Table S2.

The World Health Organization’s International Guiding Principles for Biomedical Research Involving Animals were followed during all experiments on animals. The experiments were approved by the Institutional Animal Care and Use Committee (IACUC No. 801/21).

The patient treatment protocol, blood and nasopharingeal swab samples collections, were performed in accordance with the guidelines issued by the Ethics Committee of Pediatric Research and Clinical Center of Russian Federal Medical-Biologic Agency. St. Petersburgh. The study was performed in accordance with the principles outlined in the Declaration of Helsinki.

## 3. Results

### 3.1. Hsp70 and STS Reduce LPS-Induced Increase in Production of ROS, NO, and TNFa by THP-1 Cells

In the first stage of the research, we compared the protective effects of hydrogen sulfide donor (STS) and recombinant human Hsp70 on the main mediators of inflammation induced by LPS injection. Preliminary experiments (Figure S1) showed that in the applied concentrations, these substances have no toxic effect on the viability of human model cell culture (THP-1).

**Figure 1:**
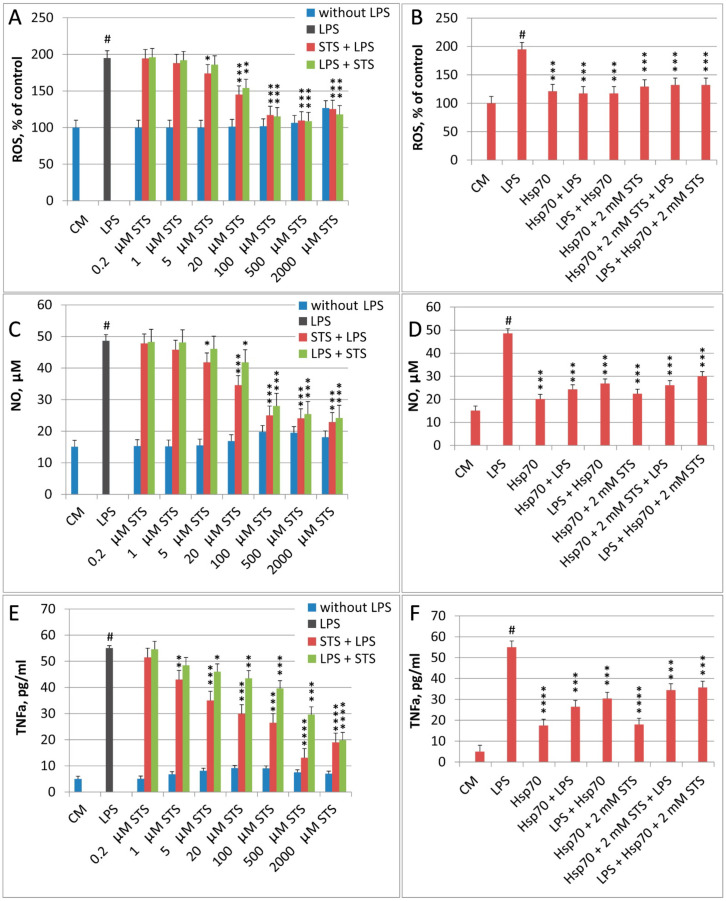
Effects of STS and Hsp70 sequential administration on LPS-induced production of ROS (**A**,**B**), NO (**C**,**D**), and TNFα (**E**,**F**). CM—culture medium; LPS-1 µg/mL LPS; Hsp70-2 μg/mL Hsp70. STS + LPS—sequential addition of first STS and then LPS; LPS + STS—sequential addition of LPS first and then STS; Hsp70 + LPS—sequential addition of first Hsp70 and then LPS; LPS + Hsp70—sequential addition of LPS first and then Hsp70; Hsp70 + 2 mM STS—joint addition of Hsp70 and 2 mM STS; LPS + Hsp70 + 2 mM STS—sequential addition of LPS first, then Hsp70 and 2 mM STS; Hsp70 + 2 mM STS + LPS—sequential addition of first Hsp70 and 2 mM STS, then LPS. N = 6, # *p* < 0.001 versus control, * *p* < 0.05, ** *p* < 0.01, *** *p* < 0.005, and **** *p* < 0.001 versus LPS.

The addition of STS to the cells caused almost no increase in the production of ROS, NO, and TNFα (Figure 1). On the other hand, STS and Hsp70 reduced LPS-induced cell response both when used separately and together before the LPS challenge (Figure 1). The protective effect of STS and Hsp70 was more effective when these agents were added to the cells before the LPS challenge. With increasing STS concentration (when injected after LPS (Figure 1C,E), the efficiency of cell protection against NO and TNFa production under the action of LPS increased dose-dependently, reaching the maximum at STS concentration equal to 500 µM and 2 mM, respectively.). Similar results were obtained exploring GYY4137 as a hydrogen sulfide donor. The maximum protective effect was observed when GYY4137 was added at a concentration of 100–200 µM (data not shown).

It should be noted that Hsp70 itself caused some increase in the production of ROS and NO, and especially TNFa by THP-1 cells (Figure 1B,D,F).

Moreover, the efficiency of cell protection from LPS-induced activation upon addition of rHsp70 before LPS administration (Figure 1B,D,F) is at the level of protection observed after the administration of 0.1 mM STS. It is of note, that the efficiency of cell protection against LPS-induced production of ROS and NO is almost independent of the order of addition of STS and LPS to the cells at all STS concentrations. For TNFα, this is true for a concentration of 2 mM STS.

When administering Hsp70 alone and after the combined action (Hsp70 + STS), the protective efficacy is somewhat weaker than under the individual action of STS. Thus, simultaneous application of both agents does not result in a cumulative protection from the effects of LPS administration. It is also important that for Hsp70 and Hsp70 + STS variants, there are no significant differences in the production of ROS, NO, and TNFα by THP-1 cells when changing the order of addition of LPS and both applied anti-inflammatory compounds (Figure 1).

**Figure 2:**
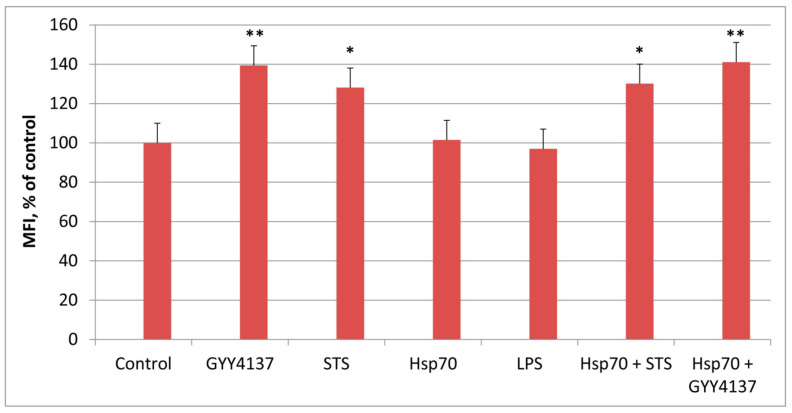
H_2_S production by THP-1 cells after administration of 200 μM GYY4137, 2 mM STS, 2 μg/mL Hsp70, and 1 μg/mL LPS. The concentration of SF7 was 0.8 μM. MFI (median fluorescence intensity) of the control was taken as 100%. N = 6, * *p* < 0.01, ** *p* < 0.005 versus control.

Since the protective anti-inflammatory effect of STS and GYY4137 is due to an increase in sulfur production, it was important to measure H_2_S levels before and after the administration of these compounds. Concentrations of STS and GYY4137 that exhibited maximal protective effect against LPS treatment (2 mM and 200 μM, respectively) were chosen for these experiments (Figure 2). It is evident from Figure 2 that STS and GYY4137 significantly increased H_2_S production while Hsp70 and LPS did not change H_2_S concentration.

To find out whether the expression level of the main genes involved in sulfur metabolism changes after addition of hydrogen sulfide donor GYY4137 to the THP-1 cells, we re-analyzed our transcriptomic data from a previously published paper [29] (Figure S2). Transcriptomic data demonstrate that the cystathinine beta synthase (*cbs)* gene is not expressed in THP-1 cells both under control conditions and after all the studied exposures. Expression levels of the cystathionine gamma-lyase (*cse*) and mercaptopyruvate sulfutransferase (*mst*) genes are very low. The expression level of *mst* gene increased slightly after GYY4137 treatment, while the expression level of *cse* was not changed (Figure S2).

### 3.2. The Administration of Hsp70 and STS Reduces the LPS-Induced Increase in TLR4 Levels in THP-1 Cells

Besides, the induction of major inflammatory mediators, an important consequence of LPS administration is the induction of TLR4 receptor expression in macrophages [42,43]. In this study, we analyzed the effects of Hsp70 as well as “fast” (GYY4137) and “slow” (STS) donors of H_2_S on TLR4 receptor expression in the cells studied. Our experiments demonstrated a significant decrease in the LPS-induced expression of TLR4 receptors in THP1 cells upon administration of both used hydrogen sulfide donors, as well as Hsp70 when added to cell culture after LPS injection (Figure S3).

Our results indicating the anti-inflammatory role of H_2_S donors on various consequences of LPS challenge in vitro, as well as data of other authors describing the protective effects of H_2_S in several models of pulmonary inflammation [17,44,45,46,47,48] encouraged us to initiate studies on the action of STS in our model of LPS-induced lung inflammation in rats.

### 3.3. STS Inhalation Exhibits Clear-Cut Protection in the Rat Model of Acute Respiratory Distress Syndrome (ARDS)

The model presented here produces direct acute lung injury which exhibited the general pathophysiological processes of ARDS. The method is based on selective intra-bronchial administration of LPS through a catheter of the main stem bronchi, followed by hyperventilation for 20 min as described in Materials and Methods. As a result, alveolar edema and lung tissue infiltration were induced. The model helps to determine the efficacy of putative agonists, including specialized proinflammatory agents, providing the basis for the development of new therapeutic approaches for patients with ARDS. Male Wistar rats aged 11–12 weeks were used in the experiment. Three groups of animals were modeled for ARDS by intratracheal injection of LPS (Materials and Methods). The degree of lung tissue injuries was determined based on pathology nomenclature (Table S3). Sodium thiosulfate—STS or solvent (physiological sodium chloride solution) was administered by inhalation according to the group affiliation (Table S1) of Materials and Methods. The effect of H_2_S on the level of IL-6, which is a major mediator of inflammation in COVID-19, was examined, as the level of this interleukin is a reliable indicator of disease severity and the patient’s chances of survival [5]. All samples were analyzed in one assay.

**Figure 3:**
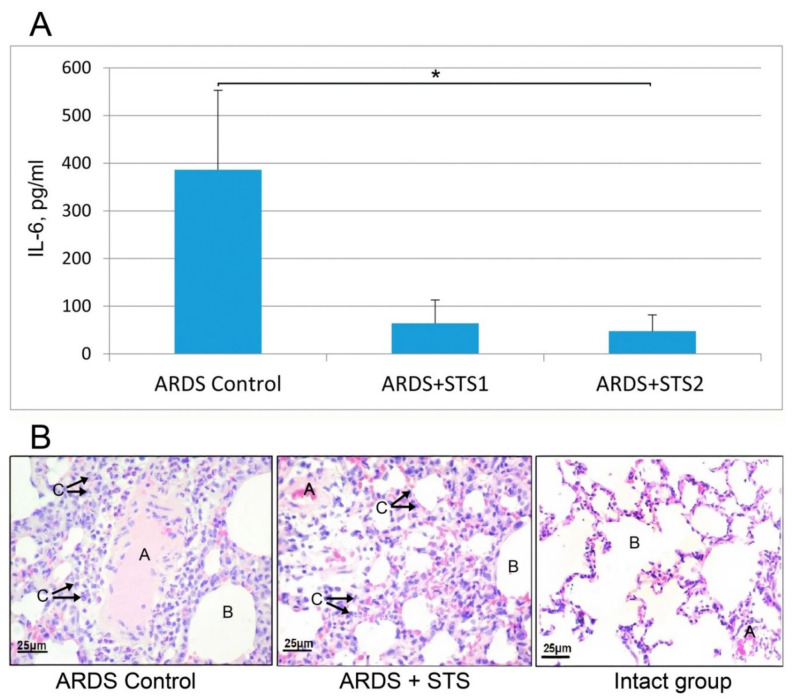
Protective effects of STS inhalations in the case of LPS-induced ARDS. (**A**) Interleukin-6 (IL-6) levels of experimental groups in serum 7 days following the initiation of ARDS. * *p* ≤ 0.05 One-way ANOVA LSD-post hoc test, mean ± SEM. (**B**) Histological examination of the lung structure in the experimental animals after LPS challenge (non-treated by STS), after STS treatment 7 days following the initiation of ARDS and lung tissue from an intact animal. Staining with hematoxylin and eosin, magnification 400×. A—vessel duct; B—intraalveolar space; Arrows C indicate infiltration of neutrophils.

The performed experiments clearly demonstrated that STS significantly ameliorated the lipopolysaccharide-induced interleukin-6 elevation in the serum in both groups of rats where STS was administrated either before or after LPS challenge (IL-6 (pg/mL), 386 ± 167 ARDS control (*n* = 4) includes LPS-treated rats; 64 ± 25 ARDS+STS1 group (*n* = 5) includes rats treated with STS before LPS, 48 ± 14 ARDS + STS2 group includes rats treated with STS after LPS challenge (*n* = 5), Figure 3A.

Histological examination of LPS-treated animals (ARDS control) revealed severe tissue injury and inflammation at the organ and the cellular level with perivascular infiltration by segmented neutrophil leukocytes and marked alveolar edema and neutrophil infiltration 7 days after injury (Figure 3B). Administration of STS attenuated the infiltration into the lungs. The semi-quantitative analysis of the lung sections by lung injury score demonstrated that ARDS was ameliorated by STS treatment (Figure 3B). For comparison, a section of the control intact lung tissue is shown in (Figure 3B).

The morphological features according to which the lung tissue in the experimental groups was microscopically evaluated are listed in Table S3. A 4-point scale was used to assess the severity of histological changes (Table S3). In all groups, there were marked capillary sludges (intra-alveolar septa with dilated capillaries containing erythrocytes) and infiltration with macrophages. At the same time in the LPS-treated group compared with the animals receiving thiosulfate, there was a significantly greater area of lung tissue lesions and pronounced infiltration of lung tissue with segmented leukocytes (Figure 3B). Histological analysis demonstrated that STS administration significantly improved lung structure (Figure 3B) and decreased the area of lung damage observed in LPS-treated animals.

### 3.4. The Analysis of the Antifibrotic Effect of Hsp70 in the Model of LPS-Induced Pneumonia in Rats

**Figure 4:**
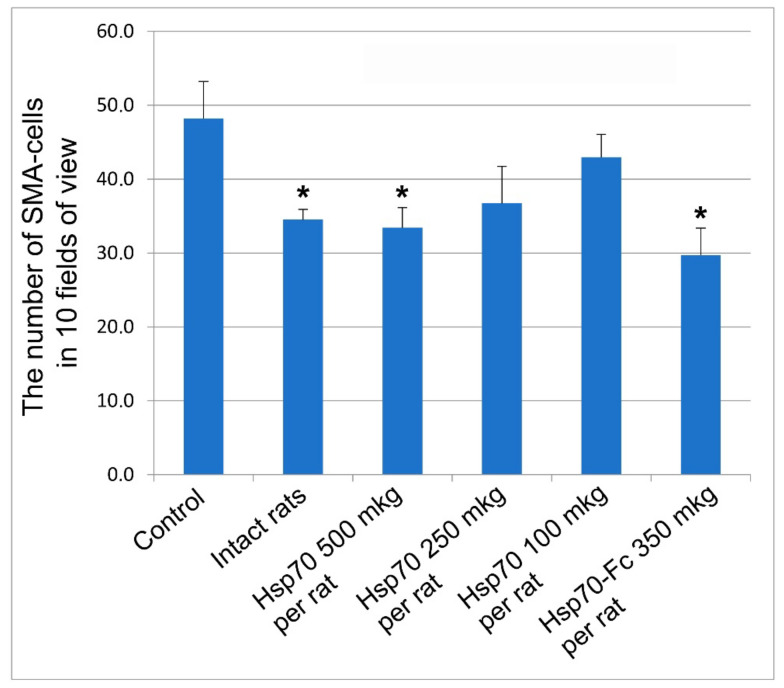
Effect of Hsp70 on the expression of alpha-SMA-positive cells in the lung stroma (in 10 fields of view) 28 days after intratracheal administration of LPS. Control-LPS-treated rats without Hsp70 administration. To monitor dose dependence of recHsp70 we used 100, 200, and 500 mg of this compound per animal (rat). Preparation of recHsp70 with prolonged effect (Hsp70-Fc) was used at 350 mg/rat taking into account its pharmacokinetics.). *—differences are statistically significant compared to the control group at *p* ≤ 0.05.

Using the model of LPS-induced pneumonia we investigated the protective properties of recombinant Hsp70 preparations. In these experiments, we evaluated the expression of alpha-smooth muscle actin (α SMA), which is an indicator of early signs of fibrosis occurring in various mammalian tissues, including lung tissue. The results of the study are presented in Figure 4.

As shown in Figure 4, the level of α-SMA expression was low in the lung tissue of intact rats. During the development of LPS-induced pneumonia, a sharp increase in α-SMA expression was observed, indicating the initiation of fibrosis in the lungs. Hsp70 administration dose-dependently decreased the expression of α-SMA, a protein that indicates the initial manifestations of lung tissue fibrosis. Our experiments demonstrated that although both recombinant protein preparations effectively reduced the expression level of α-SMA, the greatest effect was observed with two injections per week (seven injections in total) of the more stable preparation, Hsp70-Fc. Thus, our experiments showed that rHsp70 is a promising therapeutic agent capable of preventing the development of pulmonary fibrosis in pneumonia. In the next stage, we investigated the protective role of rHsp70 using a model of acute pneumonia in mice induced by a pathogenic influenza virus.

### 3.5. Protective Role of Recombinant HSP70 in Influenza Induced Pneumonia in Mice

**Figure 5:**
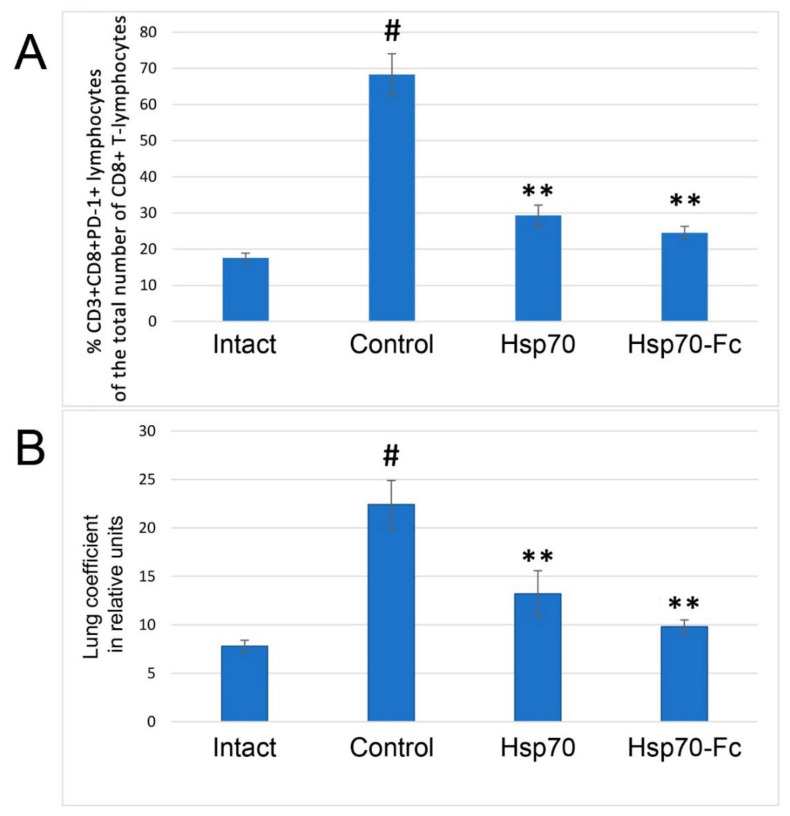
The effect of rHsp70 preparations on the level of CD+ T lymphocytes with inhibitory PD-1 receptors in peripheral blood (**A**); and pulmonary coefficient (**B**) was observed in the case of influenza-induced pneumonia in mice. # *p* < 0.005 versus intact, ** *p* < 0.01 versus control.

Our studies of the protective role of recombinant Hsp70 in the model of pneumonia caused in mice by highly pathogenic influenza virus (H3N2) showed that rHsp70 prevents the development of respiratory distress syndrome and pulmonary edema in influenza. A significant increase in the number of CD8+ T-lymphocytes with “inhibitory” PD-1 receptors in the peripheral blood was detected when mice were infected with this highly pathogenic influenza virus. Remarkably, both proteins used (rHsp70 and rHsp70-Fc) reduced 2.3–2.7-fold (*p* < 0.01) the number of CD3+CD8+PD-1+ T-lymphocytes in peripheral blood of mice during influenza-induced pneumonia (Figure 5). A decrease in CD3+CD8+ T-lymphocytes with “inhibitory” PD-1 receptors below 20% of the total number of CD3+ CD8+ T-lymphocytes in peripheral blood 5 days after infection (with at least 90% probability) represents a favorable prognosis of the outcome of influenza infection. Importantly, the use of rHsp70 in mice resulted in a 1.45–2.3-fold (*p* < 0.05) decrease in lung coefficient (LC). The protective effect of the more stable rHsp70-Fc was significantly higher than that of the unmodified rHsp70 judging by both parameters (Figure 5A,B).

The studies demonstrated the protective role of both protein preparations, the administration of which significantly (more than twice) increased the survival of tested animals with pneumonia (data not shown).

The data accumulated in the above-described experiments as well as abundant literature describing anti-inflammation effects of H_2_S and recombinant Hsp70 motivated us to investigate the role of these compounds in the case of SARS-CoV-2 infection in humans.

### 3.6. The Application of STS to Control SARS-CoV-2 Replication in Culture Cells

**Figure 6:**
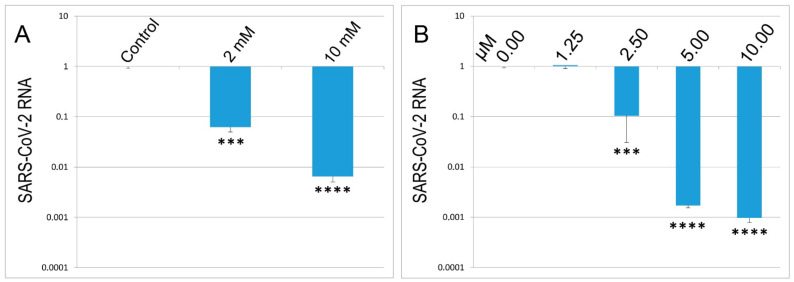
(**A**,**B**) H_2_S donors suppress replication of SARS-CoV-2 in vitro. Vero E6 cells were seeded on 6-well plates, infected with the virus, which was removed 2 h later simultaneously with the addition of STS (**A**) or GYY4137 (**B**). SARS-CoV-2 RNA levels in a conditioned medium were quantified by RT-qPCR at 4 d.p.i. *** *p* < 0.005 and **** *p* < 0.001.

In the first stage, we decided to check whether STS affects the replication of SARS-CoV-2 in Vero E6 cells. It was done as described in Materials and Methods. The compound was added to the cells 2 h post-infection, and virus replication levels were assessed by RT-qPCR 4 days later. The results are presented in Figure 6. Indeed, STS at 2–10 mM concentration decreased levels of SARS-CoV-2 RNA by 1000 times (Figure 6A). Then, a similar experiment with GYY4137, a slow donor of H_2_S, was performed. This drug also caused a strong concentration-dependent inhibition of virus replication (Figure 6B). 

The performed experiments demonstrated highly efficient inhibition of replication of the virus resulting from both H_2_S donors administration. Furthermore, both agents applied exhibited a clear-cut concentration-dependent effect on the virus replication. Notably, rHsp70 failed to modulate virus replication in our experiments (data not shown). In the next stage, we decided to investigate various effects of STS-based inhalations in COVID-19 patients.

### 3.7. The Investigation of STS Protection in COVID-19 Patients

In this section of our paper, we tried to translate the promising experimental finding of STS and Hsp70 anti-inflammatory effects described above into clinical practice. The control and experimental groups were comparable in terms of age, sex, accompanying illnesses and timing of treatment initiation and termination after the disease was revealed. Experimental and control groups were epidemiologically compatible.

Emergency early treatment of COVID-19 included the use of hot helium–oxygen breathing mixtures in combination with inhalation of sodium thiosulfate (see Materials and Methods), with the rate of coronavirus infection decreasing to 4.8% (3/69 people) compared with an infection rate of 13.8% (11/89 people) in people who did not use this early therapeutic procedure.

Patients who tested positive for SARS-CoV-2 (18 people) were also treated with a combination of hot helium–oxygen breathing mixtures and inhalation of sodium thiosulfate. The course of the disease was asymptomatic in 11 patients, and in 7 patients the treatment significantly reduced the symptoms of infection, accelerated the onset of recovery (5–7 days), and promoted rapid elimination of the virus from the patients (within 48–72 h, according to PCR-tests).

**Figure 7:**
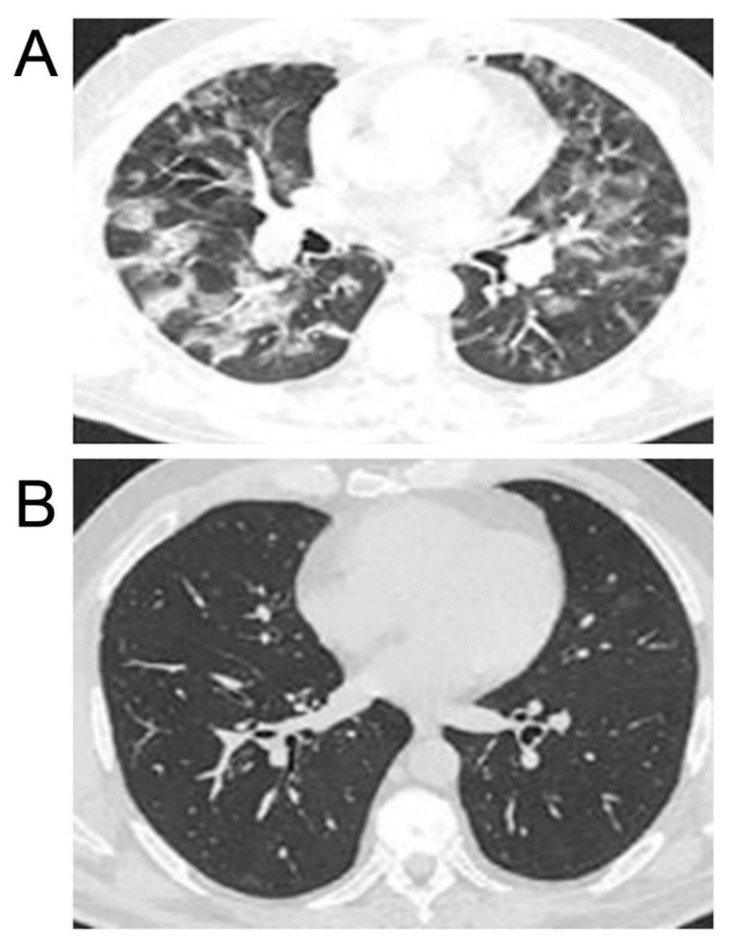
The results of computer tomography (CT) of the lungs of COVID-19 patients. (**A**) Severe case. 80% lung injury before STS treatment. (**B**) 5% lung injury after 2.5 months of application of hot helium–oxygen mixtures and inhalation of STS.

The combination of hot helium–oxygen breathing mixtures with inhalations of STS in patients with clinically severe cases with high initial lung damage, 70–85% (8 patients), resulted in full computer tomography (CT) remission of coronavirus pneumonia 45–65 days after treatment starts without respiratory failure and signs of pneumofibrosis (Figure 7). In the control group of patients (18 patients) with similar initial indices in 3 and 6 months after standard treatment, CT signs of pulmonary fibrosis (degree of lesion 45–50%) and lowered functions of external respiration (FER) (35–40%) were revealed in 12 and 8 from total 18 cases, respectively. Differences between the main group of patients (combined use of inhaled hot helium–oxygen mixtures and sodium thiosulfate) and the control group (conventional therapy) were highly significant (*p* < 0.01).

The increased level of PD-1-positive T-lymphocytes (CD3+CD8+PD-1+) in peripheral blood in COVID-19 patients indicates the development of immune exhaustion [49].

**Figure 8:**
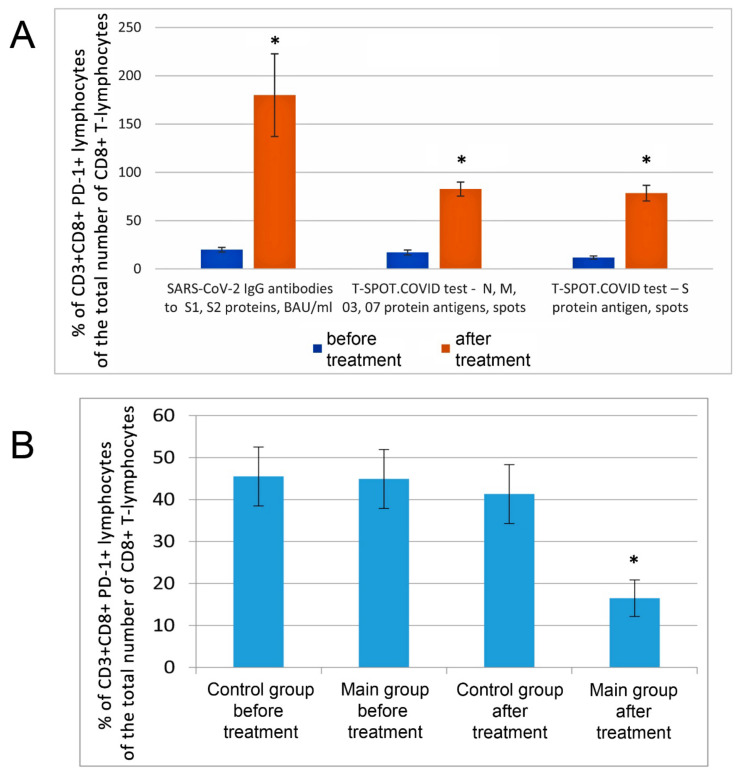
The protective effects of STS inhalation on immunity parameters of COVID-19 patients. (**A**) Comprehensive assessment of immunity (c specific antibody and T-cell response) in COVID-19 patients (n = 9) before and after treatment. (**B**) Effect of inhalation of hot helium–oxygen mixtures and STS on the number of PD-1 positive CD8+ T-lymphocytes in patients with COVID-19, % of the total number of CD8+ T-lymphocytes. “Control group”—standard therapy (8 people). Main group—a combination of inhalation with hot helium–oxygen breathing mixtures and STS (10 people—inhalations—see Table S3). *—the differences in comparison with the control group are statistically significant at *p* ≤ 0.05.

**Figure 9:**
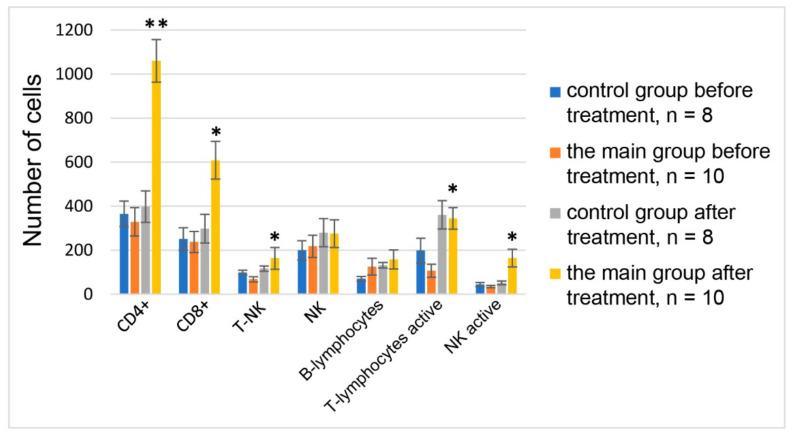
Effect of inhalation of hot helium–oxygen gas mixtures and sodium thiosulfate on the main indices of cellular immunity in COVID-19 patients. Control group—COVID-19 patients treated with standard anti-virus therapy. Main group—COVID-19 patients treated with hot oxygen–helium mixes and STS inhalation. *—the differences in comparison with the control group are statistically significant at *p* ≤ 0.05, ** *p* < 0.01.

Inhalation of hot helium–oxygen mixtures in combination with STS was found to restore immune reactivity, T-cell, and innate immunity parameters, and to prevent the development of immune exhaustion after COVID-19 (Figure 8 and Tables S4–S6). Treatment resulted in a 2.8–4.5-fold decrease in the number of lymphocytes with exhaustion markers (PD-1 receptors), an increase in acquired immunity number of T-killers (CD3+CD8+) by 3.2–5.4 times, T-helpers (CD3+CD4+) by 2.9–3.5 times, and B-lymphocytes by 1.4–1.8 (Figure 9).

The indices of innate immunity increased: the number of natural killers (NK) by 1.8–2.4 times and the number of activated NK by 2.2–2.8 times.

Thus, the proposed technology contributes to the restoration of the specific antigenic and T-cell immune response against coronavirus antigens (according to the T-SPOT^®^.COVID test) in COVID-19 patients.

Therefore, inhalation of STS in combination with hot helium–oxygen mixtures blocks the multiplication of the virus in the body, helps to restore immune reactivity, and suppresses the development of pulmonary fibrosis after infection.

## 4. Discussion

Strictly speaking, there are two complementary approaches to fighting the COVID-19 pandemic. The development of various vaccines including monoclonal ones represents the first approach which requires huge amounts of money and efforts of medical personnel. Although multiple COVID-19 vaccines are authorized and used for the prevention of coronavirus disease, no existing vaccine is effective against the viral variants that keep appearing all the time. Besides, serious side effects have been described for several vaccines and there is still an urgent need for better therapies to treat people who for some reason are not able to or choose not to use the vaccines. Therefore, another approach represents the development of effective drugs that should ameliorate the disease progression, reduce mortality and morbidity, and accelerate the recovery process after the disease. Although recently several groups have declared the development of several new anti-COVID-19 drugs [50,51], the need for cheap and efficient drugs with a low risk of drug resistance still exists. The novel therapeutic without deleterious side effects should be able to alleviate the disease progression at different stages. Basically, anti-COVID-19 drugs may target several major vulnerabilities of SARS [52,53]. Because serine protease TMPRSS2 is involved in cleaving the SARS-CoV-2 spike protein, allowing the virus to enter the cell drugs that inhibit the protease should inhibit virus activation and disease progression [54]. The potential drugs may also block cell entry by interfering with functional host receptors responsible for virus penetration into host cells [25,55]. Furthermore, the drugs may interfere with viral replication by acting on RNA-dependent RNA polymerase and prevent the escalation of inflammation to potentially lethal cytokine storm often resulting in lethality [51]. Finally, there is an urgent need for drugs that will be able to combat the consequences of the infection as NETosis (neutrophil extracellular traps) and fibrosis processes are often observed in the lungs of the COVID-19 patients [50,56,57].

Therefore, the main goal of our investigation was to develop a safe and inexpensive therapeutic approach to cure COVID-19 patients. Based on our previous results and relevant data from the literature we decided to check the protective properties of sodium thiosulfate (STS) which is already a clinically viable H_2_S donor approved by the FDA, and is also currently in clinical trials with several other H_2_S donor drugs for cardiovascular diseases, intestinal disorders, and other pathologies [2,4,20,22,58]. Besides, in our studies, we used a human recombinant major stress protein (rHsp70) with well-described anti-inflammatory properties and an important role in proteostasis [6,7,59].

In the course of our analysis, we performed a series of experiments exploring these compounds (STS and Hsp70) and various in vitro and in vivo assays to monitor the anti-inflammatory and other protective effects of these structurally and functionally different agents. The comparison of anti-inflammatory effects of STS and recombinant Hsp70 in LPS-treated THP-1 cells demonstrated a very similar protective action of both compounds (Figure 1). Interestingly, both agents exhibited significant protection and efficiently inhibited the production of all major mediators of inflammation when applied either before or after LPS-challenge. However, the effect was generally more pronounced when the compounds were applied before LPS. We also failed to observe any cumulative protective effect of STS and Hsp70 administration in the series where both agents were introduced simultaneously. A similar pattern of STS and rHsp70 effects observed in these experiments enable us to suggest that both agents exercised their anti-inflammatory and antioxidant action by exploring the same interactive signal pathways that have overlapping targets. Thus, they may both explore the reperfusion injury salvage kinase (RISK) pathway, where their signaling mechanisms converge and where they act via the regulation of nitric oxide (NO). Notably, it has been demonstrated in several studies that H_2_S can induce Hsp70 [33,34]. It was also demonstrated that both STS and rHsp70 were able to significantly decrease the LPS-induced expression of TL4 receptors in human THP1 cells (Figure S3). These results are of interest because it has been recently shown that besides the well-studied ACE2 (angiotensin-converting enzyme 2) entry receptor, which is present on only 1–2% of the cells in the lungs, several other receptors participate in the SARS-CoV-2 entry into the host cells [25]. Along this line, it was demonstrated that spike protein has a strong protein–protein interaction with TLR4 which may result in hyperinflammation [15]. It has been also recently shown that both HSP70 and H_2_S may interact with S-ACE2, AngI, and other potential receptors of SARS-CoV-2 which resulted in blocking SARS-CoV-2 entry into host cells thereby inhibiting cytokine storms and ARDS [8,55,60,61]. Recent studies have demonstrated that the binding process of ACE2 and SARS-CoV-2 S proteins is regulated by the thiol-disulfide balance of the extracellular environment. Thus, the reduction of all disulfides to sulfhydryl groups completely disrupts the binding of the SARS-CoV-2 spike protein to ACE2. The reducing agent N-acetylcysteine amide inhibits virus entry by degrading disulfides in the SARS-CoV-2 spike protein. Consequently, STS inhalation of people after contact with COVID-19 patients may prevent the virus entry into body cells by reducing disulfide bonds and decreasing the binding of the spike protein to possible receptors [55,62,63]. It is of note, that we failed to detect any changes in the expression levels of the three major genes involved in H_2_S production (*cbs*, *cse*, and *mst*) in human hepatoma cells Huh7.5 after they were infected with SARS-CoV-2 (data not shown).

Our experiments exploring two different rodent models of pneumonia revealed strong anti-inflammatory effects of both compounds studied (Figure 3 and Figure 4). In these experiments, both STS and Hsp70 ameliorated the consequences of LPS-challenge and acute pulmonary pneumonia and were able to restore the lung coefficient, inhibit the production of mediators of inflammation such as TNF-a and interleukins, and decrease the level of PD-1 receptors on CD8+ lymphocytes. It was shown that levels of serum interleukins and TNF-a in hospitalized COVID-19 patients were strong and independent predictors of disease severity and death [2,3]. Importantly, STS inhalation significantly decreased the area of LPS-induced damaged area of the lungs (Figure 7). The accumulated data corroborated the results of other authors demonstrating protective effects of STS and other donors of H_2_S in the experiments exploring other models of pneumonia including COVID-19 cases [4,17,52,53]. Notably, that survivors with severe COVID-19 were reported to have significantly higher H_2_S levels and mortality was significantly greater among patients with decreased H_2_S levels [55]. In the experiments with a model cell culture (Vero cells) routinely used to monitor various compounds potentially able to inhibit SARS-CoV-2 replication, we have demonstrated that fast (STS) and slow (GYY4137) donors of H_2_S were able to efficiently inhibit the replication of the virus (Figure 6). On the other hand, rHsp70 administration failed to modulate the replication of the SARS-CoV-2 in the infected Vero cells (data not shown) which is not surprising taking into consideration the dual role of Hsp70 and other chaperones at different stages of SARS-CoV-2 infection [61].

The data accumulated in this investigation coupled with the results of other groups that studied the protection role of H_2_S in the case of virus infection served as a basis for the development of the combined therapeutic approach to treat COVID-19 patients at different stages of the disease progression. This approach includes two phases. In the first phase, we applied hot oxygen–helium inhalations that apparently induced endogenous Hsp70. At the second phase we applied inhalations of STS in combination with lidocaine which ameliorates irritation of the upper respiratory tract, which occasionally happened after H_2_S inhalation. To our satisfaction, this combined procedure resulted in highly positive outcomes when applied to COVID-19 patients and allowed to cure a majority of high-risk patients without mechanical ventilation. The inhalation efficiently decreases the damaged area of the lungs in the case of severe infection (Figure 7). Besides, the applied therapy apparently ameliorates “cytokine storm” and stimulates the function of both innate and adaptive immunity (Tables S4–S6 and Figure 8 and Figure 9). Therefore, the developed combined therapy may be successfully and safely used in mild and severe COVID-19 cases to combat different consequences of the infection and avoid mechanical ventilation.

## 5. Concluding Remarks

Using cell cultures as well as various animal models of lung inflammation, we undertook a comprehensive study of the anti-inflammatory and other protective properties of sodium thiosulfate (STS), clinically approved donor of hydrogen sulfide (H_2_S), and a recombinant human stress protein (rHSP70). These compounds, so different in their properties and functions, demonstrated reliable anti-inflammatory effects in all models used in this work. Thus, the protective, anti-inflammatory effect of these agents has been shown in vivo in various animal models of lung inflammation. In particular, rHSP70 was found to prevent the development of respiratory distress syndrome in highly pathogenic influenza in mice, increase animal survival, and reduce the number of PD-1-positive T-lymphocytes in the peripheral blood. Our model experiments allowed us to develop a combined two-phase therapeutic approach for the treatment of COVID-19. This therapeutic procedure includes, in the first step, inhalation with hot helium–oxygen mixtures for induction of endogenous HSP70 and, in the second step, inhalation with STS. This approach has yielded very positive results in COVID-19 patients when applied to any stage of the coronavirus infection. STS inhalation reduced the area of lung lesions, restored overall immunity, and prevented the development of pulmonary fibrosis and immune exhaustion syndrome. Therefore, our experiments exploring various models as well as COVID-19 patients confirmed the hypothesized multi-faceted action of H_2_S and Hsp70 against SARS-CoV-2 infection, and demonstrated that exogenous administration of STS might be a relevant therapeutic approach for pneumonia of various etiologies.

## Data Availability

All data supporting results can be found in the Supplementary Materials.

## Supplementary Materials

The following supporting information can be downloaded at: https://www.mdpi.com/article/10.3390/biomedicines10092155/s1, Figure S1: Effect of Hsp70 and sodium thiosulfate (STS) on THP-1 cell viability, Figure S2: Effect of sodium thiosulfate (STS), Hsp70, and LPS on TLR4 level in THP-1 cells, Figure S3: Sample analysis of CD45+, CD3+CD8+ and CD3+CD8+ PD1+ lymphocytes in COVID-19 patient before(A) and after (B) treatment using combined hot helium-oxygen mixes and STS inhalations, Table S1: The effect of STS treatment after LPS challenge in model rats, Table S2: The comparative epidemiological analysis of main (treated) and control (intact) groups of patients, Table S3: Microscopic signs of lung tissue injuries observed in the studied groups, Table S4: Effect of inhalation of hot helium-oxygen mixtures and sodium thiosulfate on the number of CD3+CD4+, CD3+CD8+, CD3+CD8+ PD1+ cells in the blood of patients with COVID-19, Table S5: Comprehensive assessment of immunity (specific antibody and T-cell response-T.SPOT.COVID test) in COVID-19 patients (*n* = 9) before and after treatment, Table S6: Effect of inhalation of hot helium-oxygen gas mixtures and sodium thiosulfate on the main indicators of cellular immunity in COVID-19 patients.
