# Supplementary material for: The Effects of H2S and Recombinant Human Hsp70 on Inflammation Induced by SARS and Other Agents In Vitro and In Vivo
Source: Biomedicines. 2022 Sep 1;10(9):2155. doi: 10.3390/biomedicines10092155 (PMC9496158; doi:10.3390/biomedicines10092155)
Supplement: Supplementary file 1 [file biomedicines-10-02155-s001.zip › biomedicines-1825064-supplementary.pdf]

Supplementary Materials

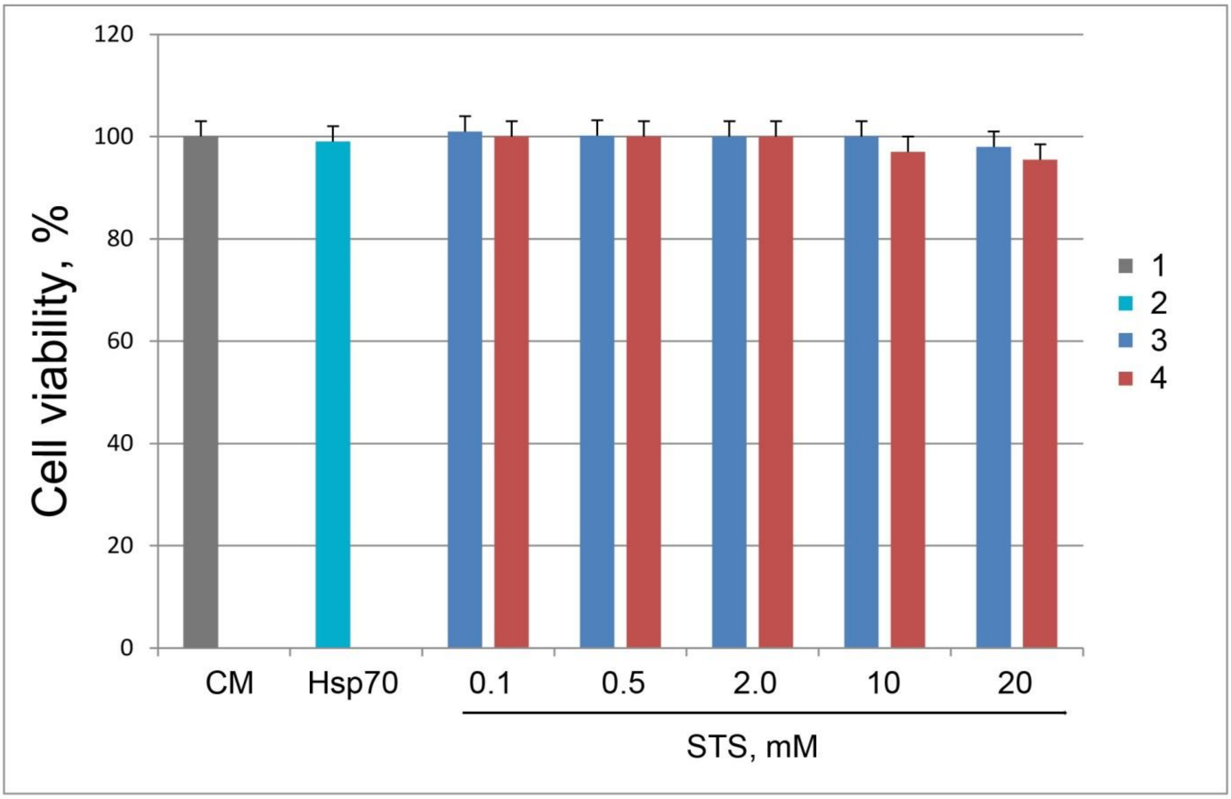

Figure S1. Effect of Hsp70 and sodium thiosulfate (STS) on THP-1 cell viability. 1 - CM - culture medium, 2 - Hsp70 - 2 μg/ml Hsp70. 3 - in the presence of STS; 4 - in the presence of Hsp70 and STS.

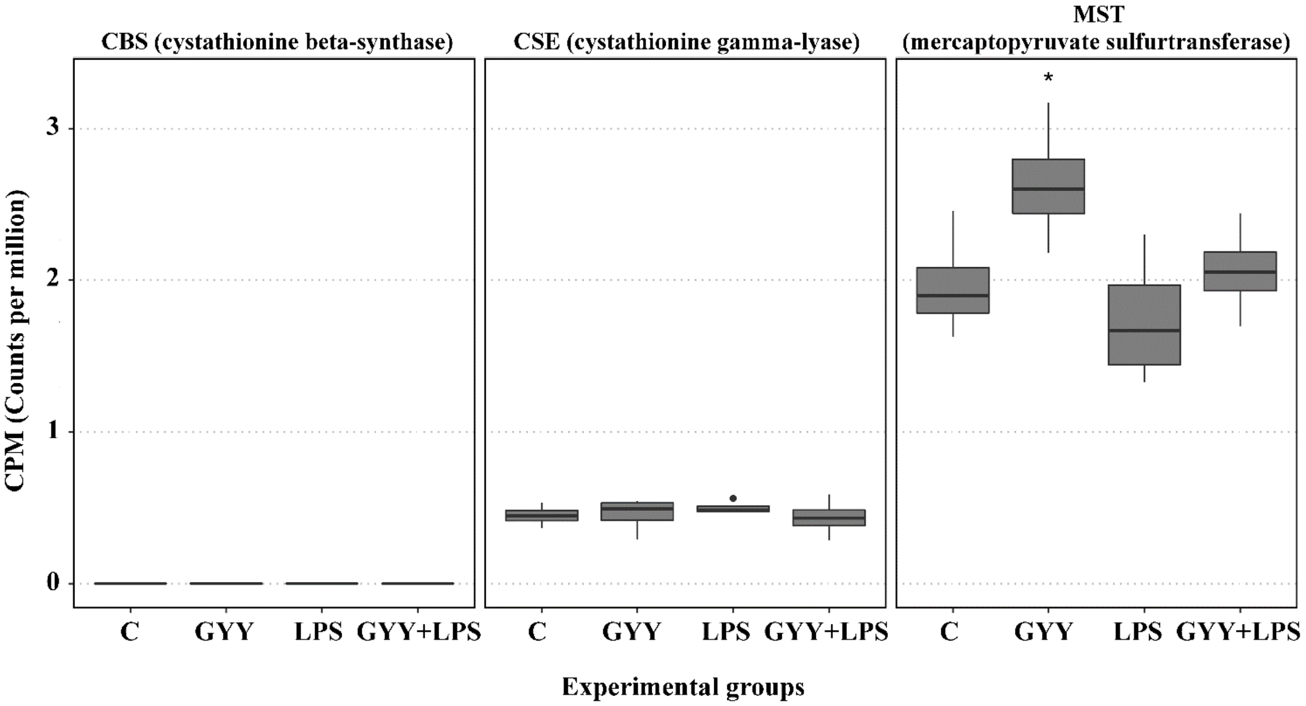

Figure S2. Expression levels of the main genes involved in H_2S production in the control (THP1) cells (C); after GYY4137 administration (GYY); after LPS challenge (LPS); and after combined action of GYY4157 added before LPS (GYY+LPS) (according to GEO accession number GSE133942 from [29]).

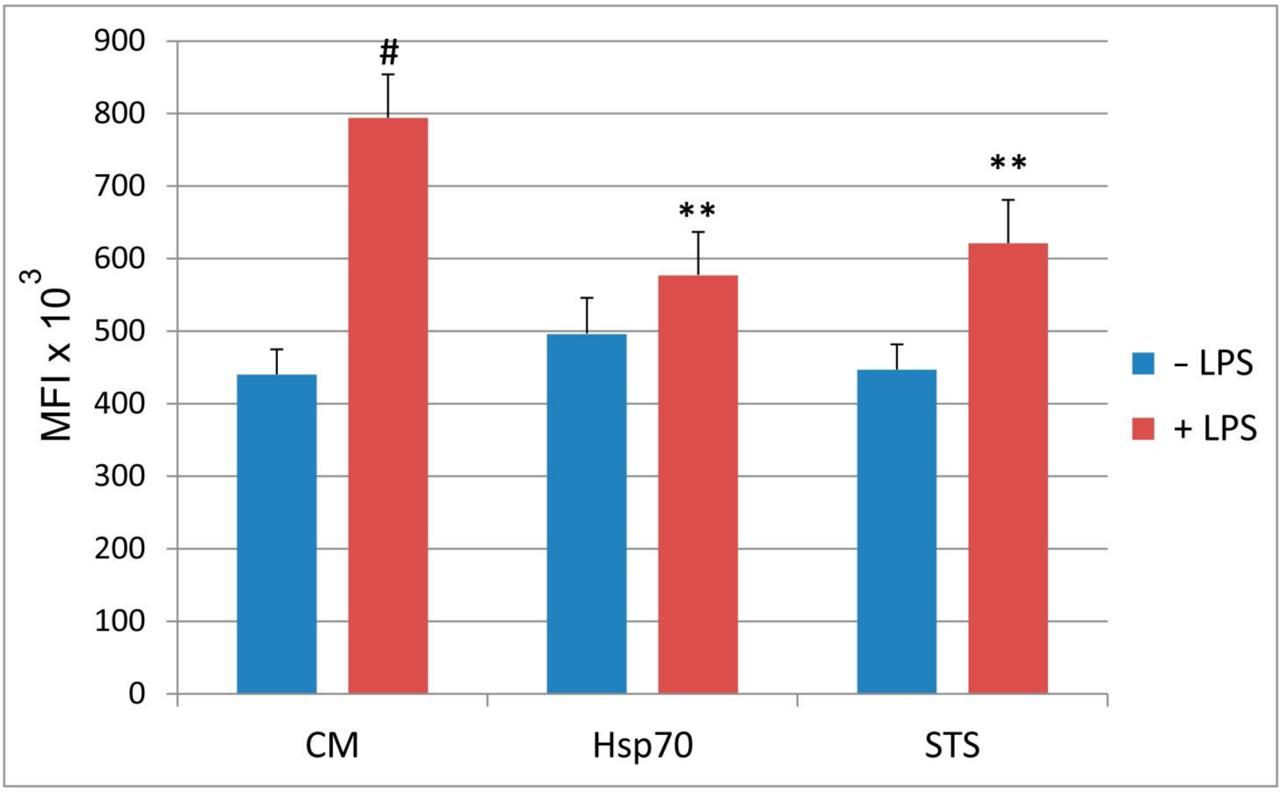

Figure S3. Effect of sodium thiosulfate (STS), Hsp70, and LPS on TLR4 level in THP-1 cells. CM – culture medium; LPS – 1 µg/ml LPS; Hsp70 - 2 µg/ml Hsp70; Hsp70+ LPS – sequential addition of 2 µg/ml Hsp70 to cells and after 60 minutes 1 µg/ml LPS; STS - 2 mM STS; STS + LPS – sequential addition of 2 mM STS to cells and after 60 minutes 1 µg/ml LPS. MFI - median fluorescence intensity. # p<0.05 LPS versus control; ** p<0.01 Hsp70 and STS versus CM+LPS.

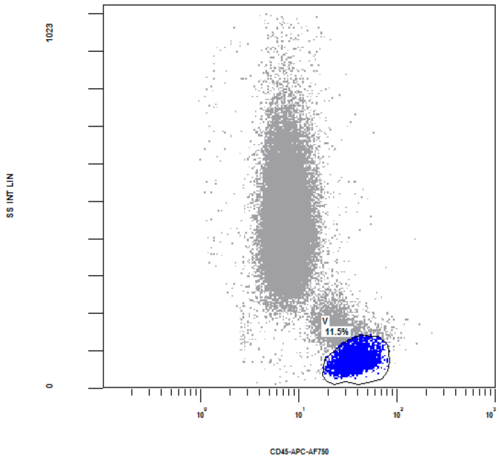

A1 x axis: CD45-APS-AF750
y axis: SS INT LIN

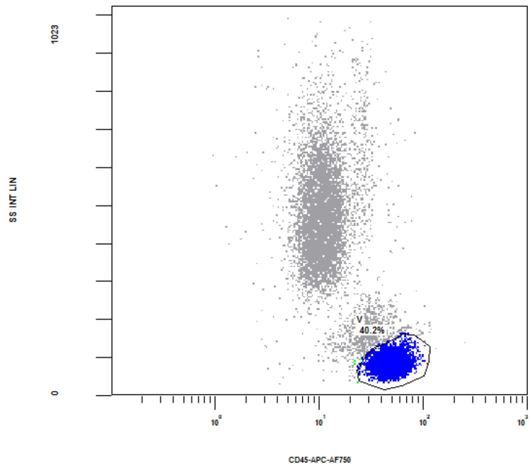

B1 x axis: CD45-APS-AF750
y axis: SS INT LIN

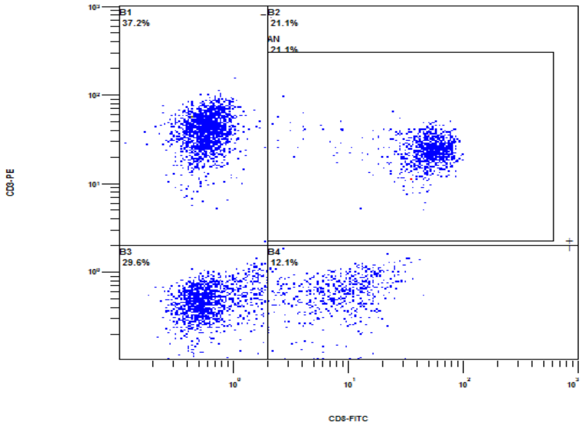

A2 x axis Cd8-FITS
y axis Cd3-PE

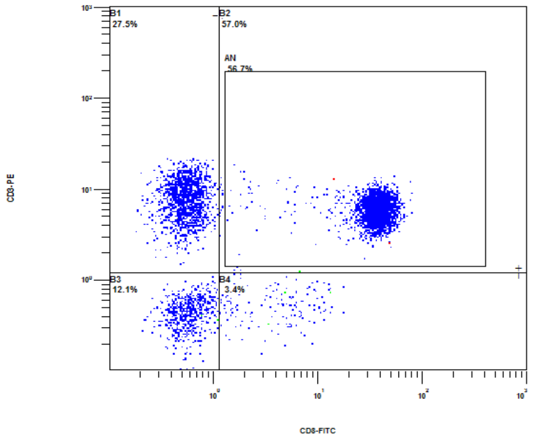

B2 x axis Cd8-FITS
y axis Cd3-PE

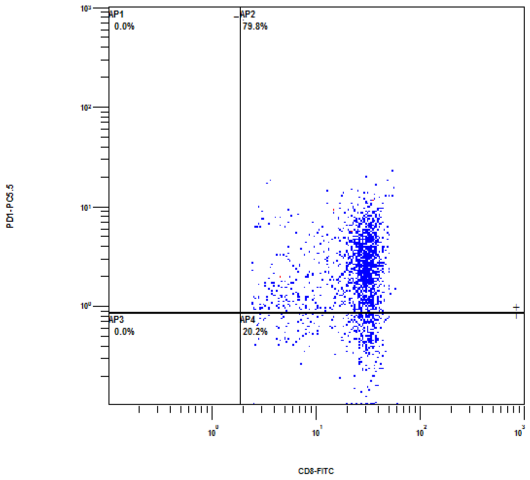

A3 x axis Cd8-FITS
y axis PD1-Pc5.5

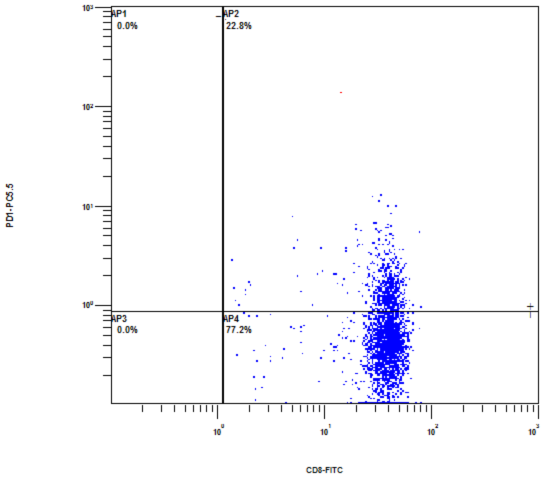

B3 x axis Cd8-FITS
y axis PD1-Pc5.5

Figure S4. Dot plots. Sample analysis of CD45+, CD3+CD8+ and CD3+CD8+ PD1+ lymphocytes in COVID-19 patient before(A) and after (B) treatment using combined hot helium-oxygen mixes and STS inhalations. The results of blood analysis by flow cytometry (Cell Lab QantaTM SC “Beckman Coulter”, USA), using antibodies of Biolegend (USA) (CD45-APC-Alexa Fluor 750; CD8-FITC; CD3-PE; PD-1-PC5.5). Before treatment lymphopenia(CD45+), decreased number of cytotoxic T-lymphocytes (CD3+CD8+) and high level of inhibitory PD-1 receptors in CD3+CD8+ T-cells were established. After therapy lymphocytosis (CD45+), increased amount of cytotoxic T-lymphocytes (CD3+CD8+) and low lewel of PD-1 receptors are evident.

Table S1. The effect of STS treatment after LPS challenge in model rats. Study design.

group no.	Name of the group	Substance tested	Mode of administration	Number of animals
1	ARDS Control	Saline solution	1 hour after LPS,<br>Day 2-7 of the study	5 (1-5)
2	ARDS + STS-1	Sodium thiosulfate	1 hour after LPS,<br>Day 2-7 of the study	4 (6-9)
3	ARDS + STS-2	Sodium thiosulfate	1 h before LPS administration,<br>Day 2-7 of the study	4 (10-13)

Table S2. The comparative epidemiological analysis of main (treated) and control (intact) groups of patients.

Indicators	Inhalation of hot oxygen-helium mix and STS (n=69)	Control untreated group (n=82)
Average age	29, 8 лет	30,4 лет
women/men	62%	67%
Smokers (Yes/No)	23/69 (33 %)	24/82 (29 %)
Comorbidities (Yes/No	12/69 (19%)	17/82 (21%)
Diabetes	1	2
Hypercholesterinemia	2	2
Hypertension	3	4
Cardiovascular	1	2
Thyroid disease	2	2
Food Allergy	1	2
Pulmonary disease	2	3
Pharmacol. treatments, Yes/No (% Yes)	10/69 (15%)	14/82 (17%)
B-blockers	2	4
Proton pump inhibitors	2	2
Hypoglycemic agents	1	2
Thyroid hormone analogs	2	2
Diuretics	4	3
Antihistamines	1	1
Mean weight (m)± SD (Min-Max)	74,6±15,6 (49-124)	76,4± 13,2 (51-126)
Mean body Mass Index)±SD (Min-Max)	25,71±5,2 (18,4-32,5)	24,9±5,5 (18,9-33,9)
Place of virus exposure (Home/Workplace	12/57	15/67
Withdrawal from the study	0	0
Side Effects	2	0
Age-years-№(%)		
<40	54(88)	70(85)
40-65 years	8(12)	12(15)

Table S3. Microscopic signs of lung tissue injuries in the studied groups

Group/Signature	**1-** ARDS control	**2-** ARDS +STS-1	**3-** ARDS +STS-2
	N=4	N=5	N=5
capillary sludges	4±0	4±0	4±0
atelectases	3±0	2,3±0,6	2,3±0,6
intraalveolar hemorrhages /diapedesis of red blood cells	0	**2±0***	**2±0***
Infiltrate with segmented leukocytes	4±0	**2±0***	**2±0***
infiltrate with macrophages	4±0	4±0	4±0
Percentage of lung tissue lesions	66,7±5,6	**36,7±11,3***	**36,7±11,3***

**P≤0.05 relative to the " ARDS control" group according to the Mann-Whitney test*

Nomenclature as in Mann PC, Vahle J, Keenan CM, Baker JF, Bradley AE, Goodman DG, Harada T, Herbert R, Kaufmann W, Kellner R, Nolte T, Rittinghausen S, Tanaka T. International harmonization of toxicologic pathology nomenclature: an overview and review of basic principles. Toxicol Pathol. 2012 40: 7S-13S. doi: 10.1177/0192623312438738. PMID: 22637736.

Table S4. **Effect of inhalation of hot helium-oxygen mixtures and sodium thiosulfate on the number of CD3+CD4+, CD3+CD8+, CD3+CD8+ PD1+ cells in the blood of patients with COVID-19.**

Patient group	Number of patients	CD3+CD4+ cells/μl	CD3+CD8+ cells/μl	CD3+CD8+PD-1+, % of total CD3+CD8+ cell number
Control group before treatment	8	364,5±58,3	251,0±51,2	45,5±7,3
The main group before treatment	10	328,2±64,3	237,4±47,9	44,9±9,6
Control group after treatment	8	397,3±71,6	297,5±64,8	41,3±6,8
Main group after treatment	10	1060,1±96,7*#	608,1±85,3*	16,5±2,4*#
Normal values		570-1100	450-850	4-20

* - differences are statistically significant in comparison with the control at p≤0,05;
# - differences are statistically significant compared with the main group before treatment at p≤0,05

Table S5. Comprehensive assessment of immunity (antigenic and T-cell response) in COVID-19 patients (n=9) before and after treatment.

Parameters	The values	
	before treatment	after treatment
SARS-CoV-2 IgG antibodies to S1, S2 proteins, BAU/ml	19,9±2,3	179,9±42,8
T-SPOT.COVID test - N, M, 03, 07 protein antigens, spots	17,0±2,6	82,6±7,2
T-SPOT.COVID test – S protein antigen, spots	11,8±1,6	78,4±8,1

Table S6. Effect of inhalation of hot helium-oxygen gas mixtures and sodium thiosulfate on the main indicators of cellular immunity in COVID-19 patients.

Patient groups	CD4+	CD8+	T-NK	NK	B-lymph	T-lymph activ.	NK-activ.
Control group before treatment, n=8	364,5± 58,3	251,0± 51,2	98,8± 10,3	199,1±44,2	69,8±10,6	198,3±56,4	44,2±8,9
Main group before treatment,n=10	328,2± 64,3	237,4± 47,9	67,3± 12,4	217,4±50,9	125,2±38,4	106,5±29,6	34,2±5,2
Control group after treatment, n=8	397,3± 71,6	297,5± 64,8	116,3± 11,7*	279,3±63,2	131,1±12,4	360,4±64,3	51,1±8,6
Main group after treatment, =10	1060,1± 96,7*	608,1± 85,3*	163,2± 49,4*	274,7±62,1	157,7±43,1	343,7±48,7*	163,6±40,6*

* - differences are statistically significant compared to the control group at $p \leq 0.05$

Supplementary Methods

Histological studies of lung tissues after LPS-STS treatment. After the animals were euthanized, the lungs were extracted and filled with a 10% neutral formalin solution. The tissue specimens were rinsed in running water, dehydrated in an ascending alcohol series, and embedded in paraffin. Then, 4–5 μm paraffin sections were stained with hematoxylin and eosin and examined by ordinary light microscopy using Leica DM1000. Microphotographs of the histological sections were made with UCMOS14000KPA, 14MP 1/2.3" APTINA CMOS sensor, and the software QuPath v.0.3.0 Histological examination included the assessment of the following morphological characteristics: capillary sludges, atelectasis, intraalveolar hemorrhages, infiltration of segmented leukocytes, infiltration of

macrophages cells and the percent of tissue damage. The extent of different inflammatory manifestations in the lungs was evaluated using a semiquantitative scoring scale: 0—none (within the normal range); 1—minimal; 2—mild; 3—moderate; 4—severe, tissue alterations are noticeable, but there is a potential for an increase in severity; 5—very severe, the maximal extent of alterations, characterizing the total organ injury.

Immunohistochemical studies of lung tissues after LPS-challenge and Hsp70 treatment.

On the 28th day, the animals were removed from the experiment by decapitation. For the pathomorphological study, lung tissue samples were placed in 10% neutral paraformaldehyde solution (Sigma) prepared on 0.01 M phosphate buffer with pH=7.4 (Sigma). Retention time in the fixative was 1-3 days at 4°C. An immunohistochemical study with the determination of antibodies to α-SMA smooth muscle actin was performed. An immunohistochemical study was performed using NovolinkTM Max Polymer Detection System (Leica Biosystems, UK) and α-SMA monoclonal antibodies (clone1A4-RTU) from DAKO (Denmark). The reaction was manifested with diaminobenzidine. The result of the positive reaction was assessed as brown staining of α-SMA positive cells in the lung stroma. The semi-quantitative assessment was performed under a Leica DM 200 microscope at a magnification of 400 (40×10) in 10 fields of view.
